# EMPOWERING PEOPLE OF ALL AGES BY PROMOTING POSITIVE VIEWS OF AGING AMONG PK-12 STUDENTS

**DOI:** 10.1093/geroni/igad104.1127

**Published:** 2023-12-21

**Authors:** Tina Newsham, Lisa Borrero

## Abstract

Beliefs about aging and older adults form early in life and persist. People who hold positive views of aging rather than internalized ageism live, on average, 7.5 years longer. Communities where older adults retain meaningful engagement and are respected experience greater longevity and better social and physical well-being among people of all ages. Having positive interactions with older adults increases likelihood of pursuing careers working with older people. Promoting positive, accurate views of aging benefits individuals (of all ages) but is most effective when implemented early in life. Children, however, are rarely intentionally taught about aging and learn primarily negative stereotypes through media such as cartoons, books, and fairy tales. In this symposium, presenters will share projects aimed at disrupting the perpetuation of ageist stereotypes through intentional work with young children to promote positive views of aging. Laura Donorfio will present an aging education project developed for the Connecticut school system via an Administration on Aging grant. Next, Elizabeth Bergman will present two intergenerational case study projects involving elementary school-aged children, college students, and elders. Edward Miller will then the describe and sharing findings from an ongoing intergenerational tutoring intervention, begun during the early stages of the COVID-19 pandemic, wherein older adults provide free, online evidence-based tutoring to bi-lingual kindergartners. Finally, Tina Newsham and Cynthia Hancock will share research on a toolkit developed for preschool-2nd grade teachers to introduce information about aging and centenarians as a way to celebrate the 100th day of school. Lisa Borrero will serve as discussant. This is an Intergenerational Learning, Research, and Community Engagement Interest Group Sponsored Symposium.

